# Supplementary material for: Transcriptomic Insights into Mechanisms of Early Seed Maturation in the Garden Pea (Pisum sativum L.)
Source: Cells. 2020 Mar 23;9(3):779. doi: 10.3390/cells9030779 (PMC7140803; doi:10.3390/cells9030779)
Supplement: Supplementary file 1 [file cells-09-00779-s001.zip › Supplementary materials/FileS3.zip!/FileS2/Hormones - Results/Summary Sheets - Modules/D-Cluster/Profiles.pdf]

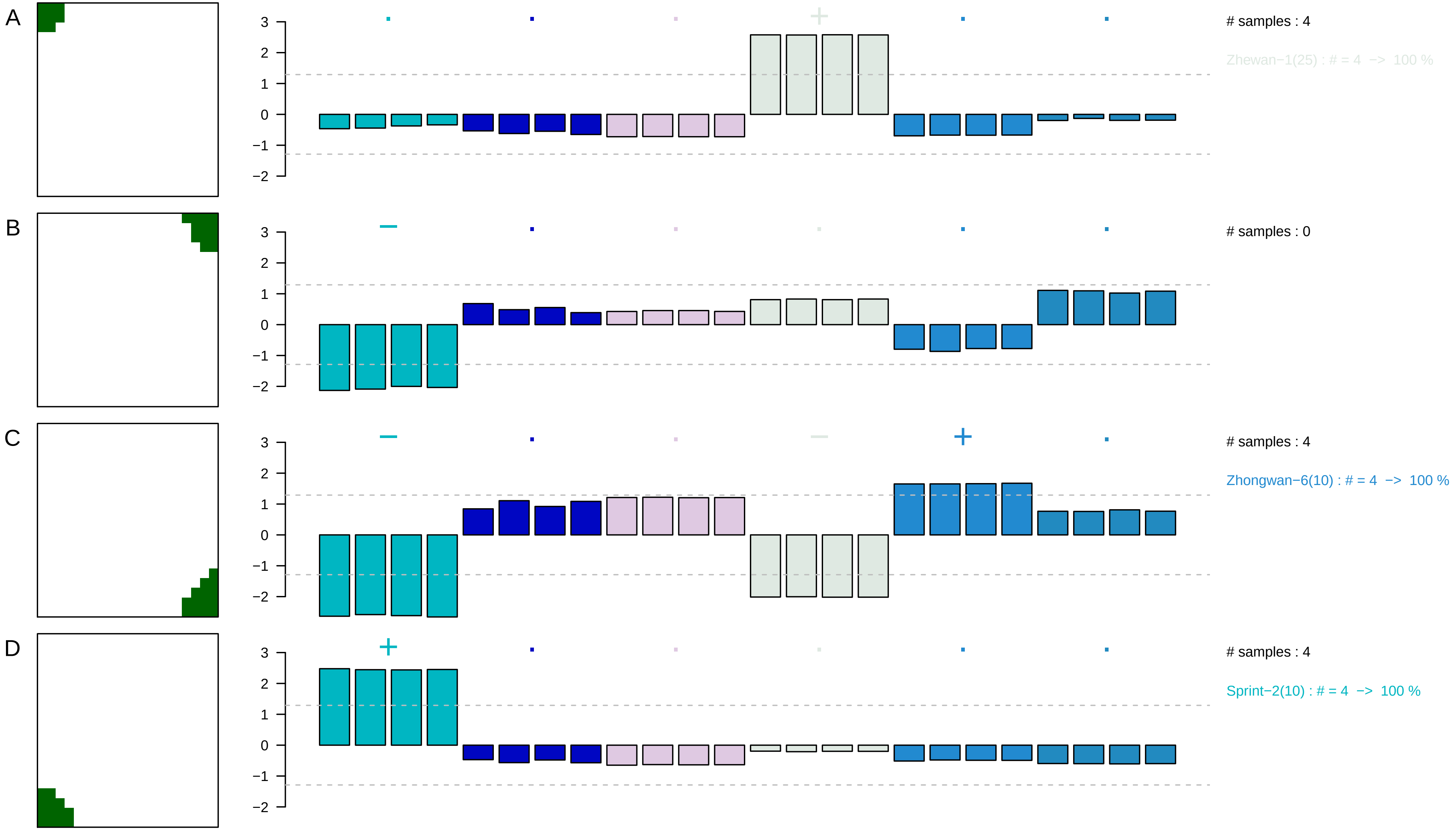
A
B
C
D
3
2
1
0
−1
−2
samples : 4
Zhewan−1(25) : # = 4 −> 100 %
samples : 0
samples : 4
Zhongwan−6(10) : # = 4 −> 100 %
samples : 4
Sprint−2(10) : # = 4 −> 100 %

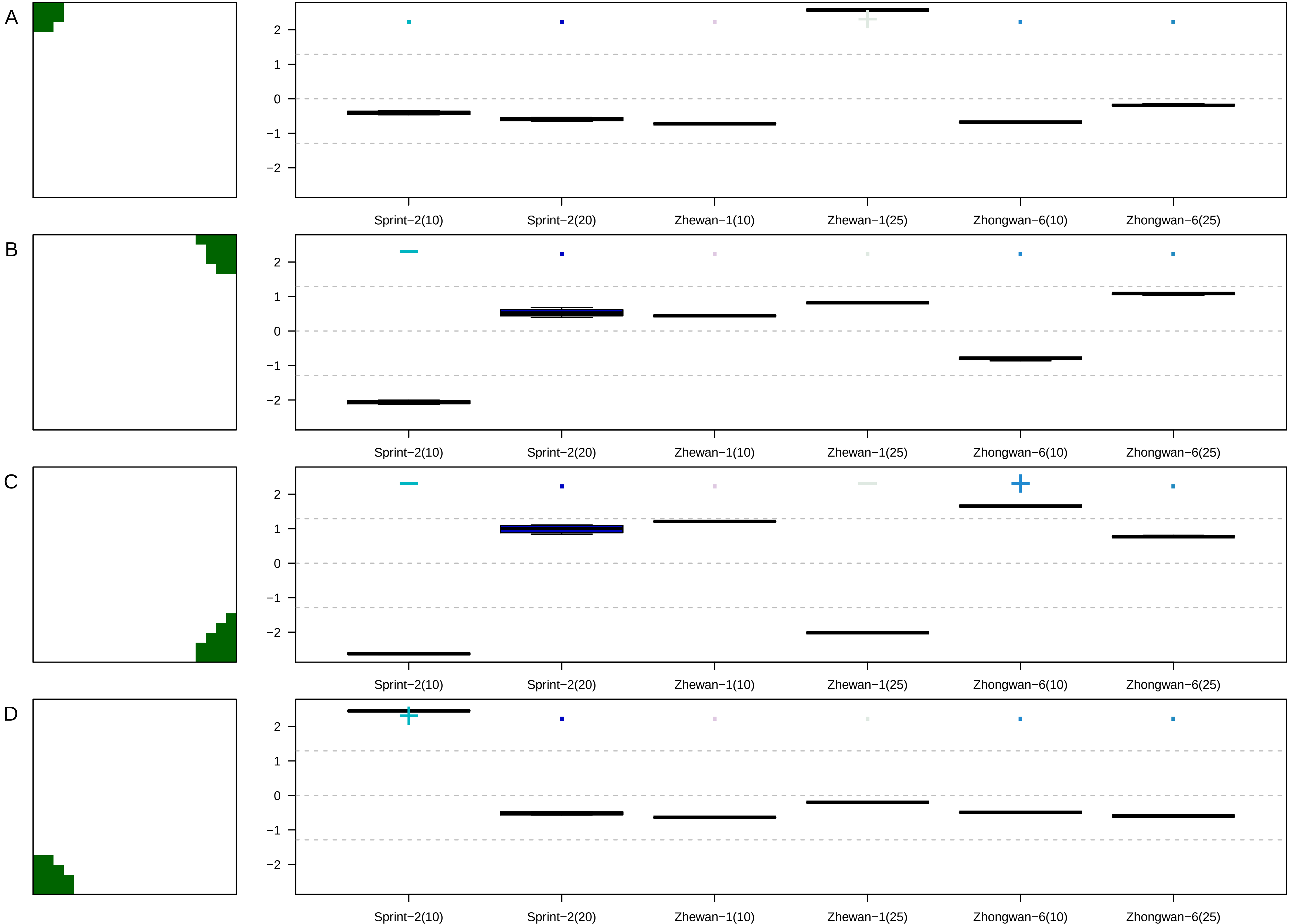
A
B
C
D
2
1
0
−1
−2
Sprint−2(10)
Sprint−2(20)
Zhewan−1(10)
Zhewan−1(25)
Zhongwan−6(10)
Zhongwan−6(25)
